# Morphological and Ultrastructural Characterization of Hemocytes in an Insect Model, the Hematophagous *Dipetalogaster maxima* (Hemiptera: Reduviidae)

**DOI:** 10.3390/insects12070640

**Published:** 2021-07-14

**Authors:** Natalia R. Moyetta, Fabián O. Ramos, Jimena Leyria, Lilián E. Canavoso, Leonardo L. Fruttero

**Affiliations:** 1 Departamento de Bioquímica Clínica, Facultad de Ciencias Químicas, Universidad Nacional de Córdoba, Córdoba 5000, Argentina; nmoyetta@unc.edu.ar (N.R.M.); fabian.ramos@unc.edu.ar (F.O.R.); jleyria@fcq.unc.edu.ar (J.L.); lilian.canavoso@unc.edu.ar (L.E.C.); 2 Centro de Investigaciones en Bioquímica Clínica e Inmunología (CIBICI), Consejo Nacional de Investigaciones Científicas y Técnicas (CONICET), Córdoba 5000, Argentina

**Keywords:** hemocyte, triatominae, morphology, ultrastructure, classification

## Abstract

**Simple Summary:**

Chagas’ disease is a debilitating and life-threatening disease endemic of the Americas, although it currently affects about six to seven million people around the world. The triatomines, also known as kissing bugs, are blood-feeding insects that play a key role in the transmission of Chagas’ disease since they are the vectors of the parasite *Trypanosoma cruzi*, the causative agent of the illness. On the other hand, the hemocytes are the cells present in the circulatory system of insects and other invertebrates. These cells are comparable to the white blood cells of vertebrates and fulfill vital functions in coagulation and defense against pathogens. The classification of hemocytes is mainly based in their cell shape, which is technically challenging to assess, and the authors have not always agreed upon this subject. In this study we combined different techniques to classify the hemocytes of the kissing bug *Dipetalogaster maxima* in a juvenile stage of development. We characterized the hemocytes in five types, including plasmatocytes, granulocytes, prohemocytes, adipohemocytes and oenocytes. These findings contribute to the understanding of insect and triatomine physiology and can be applied to unravel basic aspects of insect immune responses, coagulation cascades and endocrine processes.

**Abstract:**

Hemocytes, the cells present in the hemolymph of insects and other invertebrates, perform several physiological functions, including innate immunity. The current classification of hemocyte types is based mostly on morphological features; however, divergences have emerged among specialists in triatomines, the insect vectors of Chagas’ disease (Hemiptera: Reduviidae). Here, we have combined technical approaches in order to characterize the hemocytes from fifth instar nymphs of the triatomine *Dipetalogaster maxima*. Moreover, in this work we describe, for the first time, the ultrastructural features of *D. maxima* hemocytes. Using phase contrast microscopy of fresh preparations, five hemocyte populations were identified and further characterized by immunofluorescence, flow cytometry and transmission electron microscopy. The plasmatocytes and the granulocytes were the most abundant cell types, although prohemocytes, adipohemocytes and oenocytes were also found. This work sheds light on a controversial aspect of triatomine cell biology and physiology setting the basis for future in-depth studies directed to address hemocyte classification using non-microscopy-based markers.

## 1. Introduction

Kissing bugs or triatomines (Hemiptera: Reduviidae) are a subfamily of hematophagous insects with relevance in public health due to the fact that they are vectors of the protozoan parasite *Trypanosoma cruzi* (Chagas, 1909), the causative agent of Chagas disease or American trypanosomiasis [1]. Chagas disease is a debilitating and potentially life-threatening illness endemic in Latin America. Currently, the disease affects approximately six to seven million people [2]. On the other hand, *Dipetalogaster maxima* (Uhler, 1894) is the biggest triatomine and it is found in dry rocky parts of the Southern area of the California peninsula (Mexico). This species harbors epidemiological interest given its ability to inhabit peridomestic and intradomestic rural areas [3,4,5].

The hemocytes are the cells that circulate within the hemocele of insects and together with plasma and dissolved organic and inorganic molecules constitute the hemolymph, the analog of vertebrate’s blood [6]. Hemocytes perform several functions in the context of insect physiology, including humoral and cellular immune responses. In particular, these cells participate in phagocytosis, encapsulation, nodulation and coagulation, and are also involved in the metabolism, synthesis and storage of nutrients. The types of hemocytes and their abundance may vary according to the insect species as well as their developmental and physiological state [7,8,9,10]. 

Our laboratory has been using *D. maxima* as an experimental model for more than two decades, mainly focusing in biochemical and physiological aspects related to lipid and protein metabolism and reproduction [11,12,13,14]. We recently reported that the hemocytes of fifth instar nymphs express cathepsin D [15], a lysosomal aspartic endopeptidase involved in physiological functions such as reproduction [16], digestion [17] and immunity [18,19]. The hemocytes of *D. maxima* displayed heterogeneous immunofluorescence patterns for cathepsin D, a fact which might be compatible with the presence of different hemocyte types performing diverse functions [15]. 

The classification of hemocytes has relied mostly on morphological and ultrastructural characters, and controversies still exist among the authors in the classic literature regarding triatomine hemocyte types [20,21,22,23,24,25,26,27,28]. In this work, we have performed the morphological and ultrastructural characterization of the hemocytes of *D. maxima* fifth instar nymphs. We also employed for the first time in triatomines flow cytometry in an attempt to improve the classification of cell populations.

## 2. Materials and Methods

### 2.1. Insects

All experiments were conducted employing fifth-instar nymphs of *D. maxima* 7 days after a blood meal. Insects were from a colony maintained under standardized conditions [11] and fed on hen blood following the guidelines of the National Institute of Parasitology [29].

### 2.2. Hemolymph Collection

Unless otherwise stated, the hemolymph was collected from each insect under sterile conditions. Hemolymph was placed in microtubes containing a few crystals of phenylthiourea to avoid melanization. Samples were diluted with an anticoagulant solution (10 mM Na_2_EDTA, 100 mM glucose, 62 mM NaCl, 30 mM sodium citrate, 26 mM citric acid, pH 4.6) in a ratio of 1:5 *v/v* [20]. 

### 2.3. Phase Contrast Microscopy

Immediately after collection, fresh, unfixed and unstained hemocytes were analyzed by phase contrast microscopy (Axioplan Zeiss, Oberkochen, Germany). The images were registered with a CCD Camera (Micromax, Princeton Instruments, Downingtown, PA, USA) and the software Metamorph 3.0 (Universal Imaging Corp., Downingtown, PA, USA).

### 2.4. Fluorescence Microscopy

For this set of assays, the samples containing hemocytes were placed onto poly-L-lysine-treated slides. After incubation in a humid chamber for 1 h at room temperature, hemocytes were fixed with 4% paraformaldehyde for 20 min [30]. After each step, slides were washed twice with Phosphate Buffered Saline (PBS: 6.6 mM Na_2_HPO_4_/KH_2_PO_4_, 150 mM NaCl, pH 7.4) for 5 min each. Hemocytes were permeabilized and blocked in 0.1% Triton X-100, 2.5% bovine serum albumin (BSA), 5% fetal bovine serum in PBS for 1 h at room temperature. Primary and secondary antibodies were diluted in 1% BSA in PBS. All incubations were performed in humid chambers. Immunofluorescence control assays were carried out omitting one or both antibodies. Slides were incubated with the polyclonal anti-β tubulin antibody [dilution 1:200, rabbit anti-β tubulin (G8) of human origin, sc-55529, Santa Cruz Biotechnology, Inc., Santa Cruz, CA, USA] and with the anti-rabbit IgG labeled with Alexa Fluor^®^ 488 (1:300, Molecular Probes, Eugene, OR, USA) for 1 h at 37 °C each. The nuclei were stained with 300 nM DAPI for 2 min; the slides were mounted in Fluorsave (Calbiochem, Darmstadt, Germany) and observed with a Leica DMi8 microscope (Leica Microsystems, Wetzlar, Germany). 

### 2.5. Transmission Electron Microscopy (TEM)

For TEM assays, hemolymph collected from 10 insects was pooled and centrifuged at 500× *g* for 10 min at room temperature [31,32] to enrich the preparation with hemocytes without inducing visible cell damage. The supernatant was discarded and the pellet was suspended and fixed in Karnovsky mixture containing 4% formaldehyde and 2% glutaraldehyde in 0.1 M cacodylate buffer for 2 h and processed as previously described [33]. Fixed cells were washed and treated with 1% OsO_4_, dehydrated in a series of acetone solutions, and embedded in Embed 812 resin. After a 48-h polymerization stage, 200 nm semithin sections obtained using a Jeol microtome (Tokyo, Japan) were stained with toluidine blue for high-resolution light microscopy analysis. Finally, 90-nm ultrathin sections obtained in a JUM-7 ultramicrotome (Jeol, Tokyo, Japan) were placed on grids, post-stained with uranyl acetate/lead citrate solutions, examined in an electron microscope (Zeiss Leo 906-E, Oberkochen, Germany), and photographed with a Megaview III camera (Olympus, Center Valley, PA, USA).

### 2.6. Flow Cytometry

Collected hemolymph was diluted to 400 μL with the buffer *Rhodnius prolixus* saline (150 mM NaCl; 8.6 mM KCl; 2 mM CaCl_2_; 8.5 mM MgCl_2_; 4 mM NaHCO_3_; 34 mM glucose; 5 mM HEPES, pH 7, [34]). The hemocytes were stained with 300 nM 4′,6-diamidine-2′-phenylindole (DAPI) and/or 1.5 µM propidium iodide (PI) for 5 min and immediately subjected to flow cytometry. Data were acquired and analyzed in a LSRFortessa X-20 flow cytometer (Becton Dickinson, San Jose, CA, USA) and further examined using FACS-DIVA software (BD Biosciences, Franklin Lakes, NJ, USA). A flow of 12 µL/min was used, a total of 10,000 events were collected from each sample, and a threshold value of 300 in forward scatter (FSC) and 200 in side scatter (SSC) was typically applied to prevent data collection for smaller particles. Total cell counts and cell percentages were obtained by flow cytometry software. In some cases, flow cytometric analysis was applied to a specific cell type by first gating on the cell population in FSC vs. SSC plots and then determining the mean fluorescence intensity values for the selected population. In this way, fluorescence measurements and concentrations for a given cell type were determined.

## 3. Results

### 3.1. Phase-Contrast Microscopy

**Figure 1:**
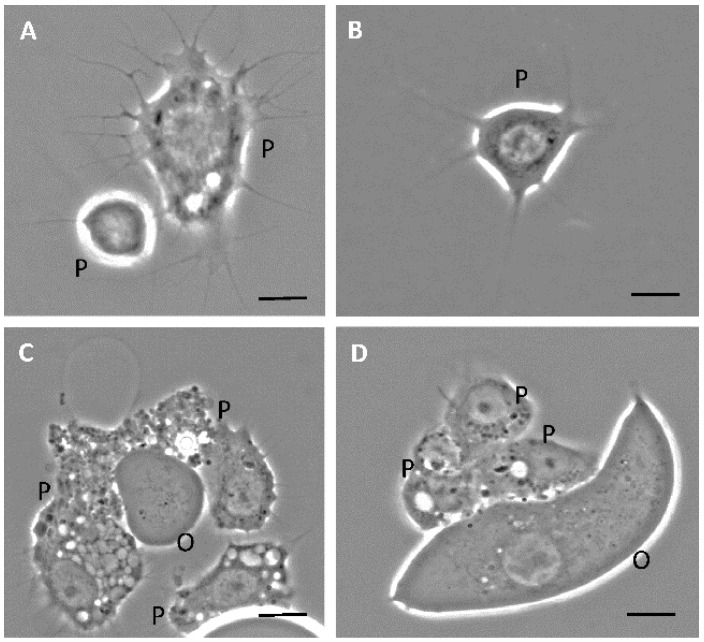
(**A**–**D**) Phase contrast microscopy of fifth instar nymphs hemocytes of *Dipetalogaster maxima*. P, plasmatocyte; O, oenocyte. Bars: 10 μm.

**Figure 2:**
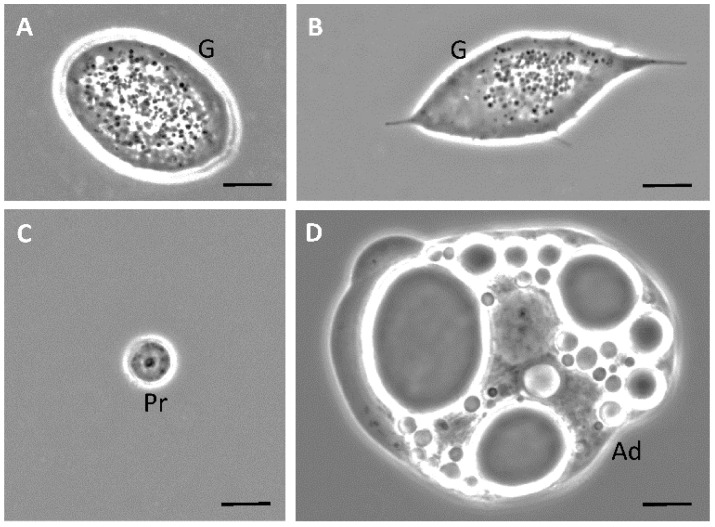
(**A**–**D**) Phase contrast microscopy of fifth instar nymphs hemocytes of *Dipetalogaster maxima*. G, granulocyte; Pr, prohemocyte; Ad, adipohemocyte. Bars: 10 μm.

**Figure 3:**
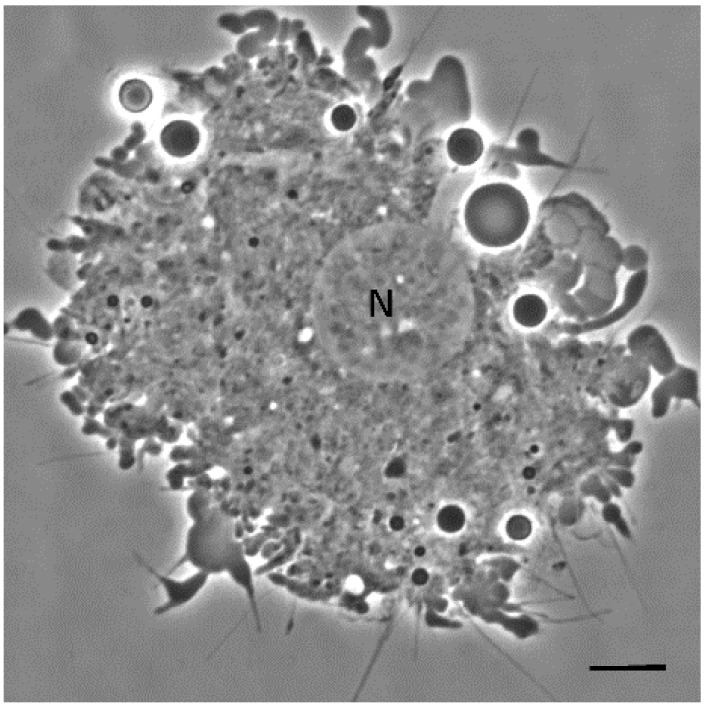
Phase contrast microscopy of a giant cell from fifth instar nymphs *of Dipetalogaster maxima*. N, nucleus. Bas: 10 μm.

The results of phase-contrast microscopy using unfixed unstained hemocytes in *D. maxima* fifth instar nymphs evidenced five cell types based on morphology: plasmatocytes and granulocytes which were the more abundant types, as well as prohemocytes, adipohemocytes and oenocytes (Figure 1 and Figure 2). It was also observed giant cells, which were very rare (Figure 3). Variability in shape and size was observed in plasmatocytes with lengths ranging from 11 to more than 60 μm and widths ranging from 6 to 25 μm. Plasmatocytes were spindle-shaped, round and/or flattened and were the commonest cell type (Figure 1). They contained different inclusions and vacuoles in the cytoplasm. Nuclei were located in a central position or rather displayed to the cell border. The cells often presented pseudopodia and were found in islets of aggregations, being the most numerous cell type present in a given aggregate (Figure 1C,D). Oenocytes were also relatively scarce and presented two subtypes: one smaller and rounded with a diameter of about 15–20 μm and the other one was larger, more than 60 μm in length, and spindle-shaped with an eccentric nucleus. Their main features were the phase-dark and homogeneity of their cytoplasm (Figure 1C,D). Granulocytes, also known as granular cells, were a very frequent type, variable in shape, from round to spindle-shaped with lengths ranging from 12 to 55 μm while the widths ranged from 12 to 24 μm. Their main characteristic was the presence of small sized granules. The nuclei were centrally located and pseudopodia were occasionally observed (Figure 2A,B). Prohemocytes were small (4–8 μm in diameter) and rounded cells with central nuclei surrounded by a thin rim of cytoplasm (Figure 2C). Adipohemocytes were scarce large cells of more than 65 μm in diameter, with large and refringent lipid droplets or adiposomes (Figure 2D). Giant cells of above 80 μm in size were even more rarely observed (Figure 3).

### 3.2. Transmission Electron Microscopy

**Figure 4:**
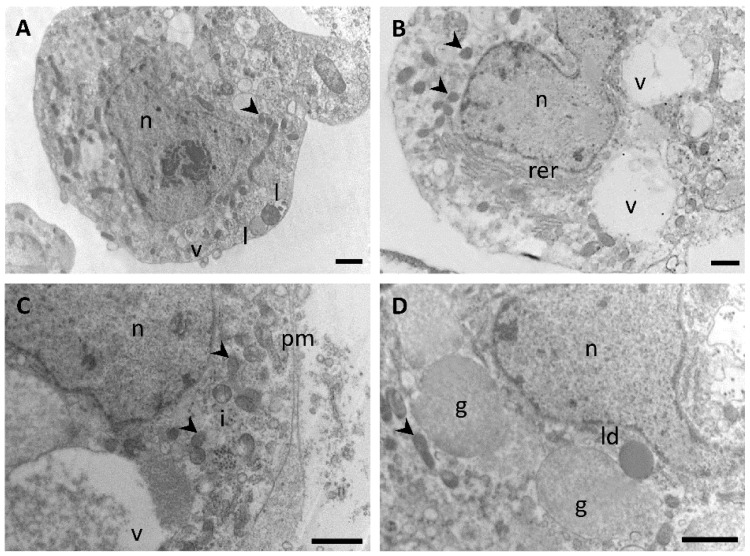
(**A**–**D**) Transmission electron microscopy of cells compatible with plasmatocytes of fifth instar nymphs of *Dipetalogaster maxima*. Arrowheads indicate mitochondria; n, nucleus; v, vesicles; i, inclusions; ld, lipid droplet; rer, rough endoplasmic reticulum; pm, plasma membrane; g, granules; l, lysosomes. Bars: 1 μm.

The polymorphism observed in plasmatocytes by phase contrast microscopy (Figure 1A–D) was also evidenced at the level of TEM examination (Figure 4). These cells presented polymorphic nuclei of round, irregular or bilobed shapes, with several patches of electron opaque chromatin. In general, the cytoplasm of putative plasmatocytes presented more diversity in organelles when compared with other types of hemocytes. Thus, plasmatocytes exhibited a wide variety of inclusions, granules and vesicles, being the latter either empty or filled with suspended amorphous substances (Figure 4A–D). Lysosomes (Figure 4A) and lipid droplets (Figure 4D) were present although they were not detected in all plasmatocytes. A well-developed perinuclear rough endoplasmic reticulum (RER) was a common feature (Figure 4B) whereas the presence of abundant mitochondria (Figure 4A–D) suggested a high rate of energy requirement in these cells likely associated with their function in capsule and nodule formation. 

**Figure 5:**
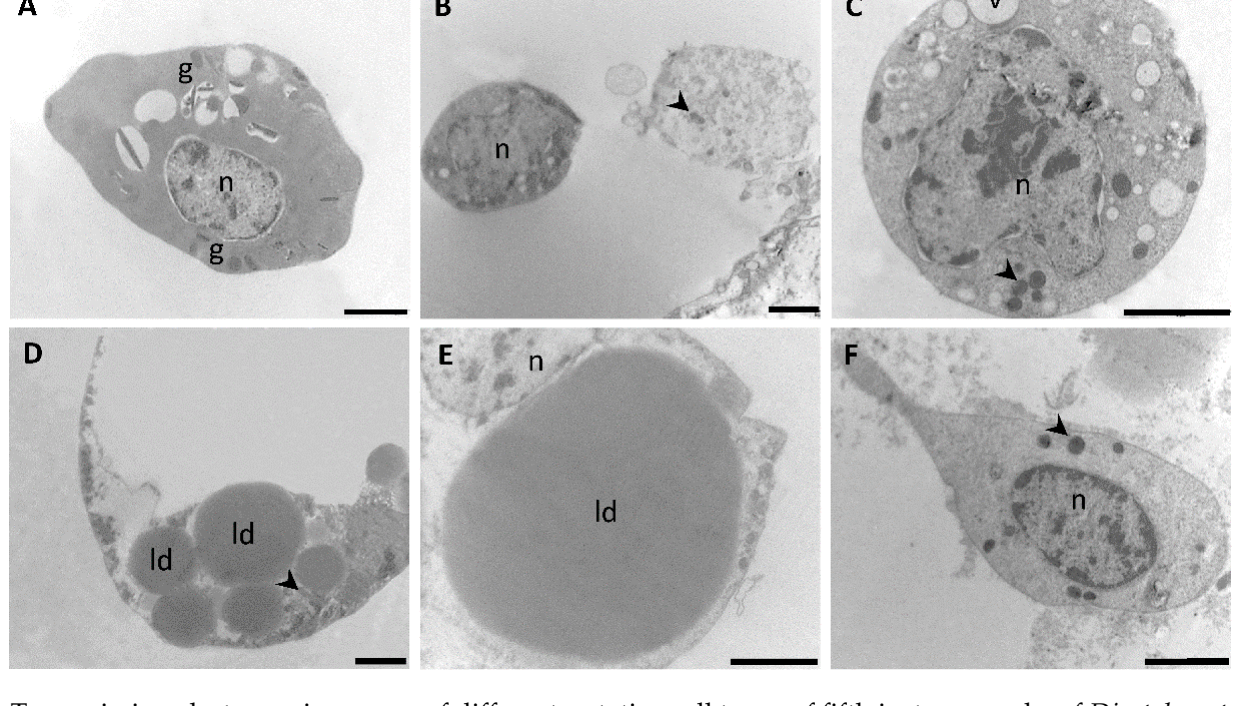
Transmission electron microscopy of different putative cell types of fifth instar nymphs of *Dipetalogaster maxima*. (**A**) granulocytes, (**B**,**C**) prohemocytes, (**D**,**E**) adipohemocytes and (**F**) oenocytes. Arrowheads indicate mitochondria; n, nucleus; ld, lipid droplet; g, granules. Bars: 2 μm for (**A**–**D**) and 5 μm for (**E**,**F**).

Cells compatible with granulocytes were comparatively simple to classify under TEM (Figure 5A). Prohemocytes displayed more variability under TEM examination (Figure 5B,C) when compared to phase contrast analysis (Figure 2C). Cells falling under the description of prohemocytes were visualized employing TEM and identified mainly because of their sizes, round shapes and large nuclei with several islands of heterochromatin. In some cells, a conspicuous nucleolus was observed (Figure 5C). In most prohemocytes, the cytoplasm presented inclusions and vacuoles (Figure 5B,C). Adipohemocytes (Figure 5D,E) showed their characteristic cytoplasm filled with lipid droplets of different electron densities and with diameters up to 15 µm. It was also observed that abundant inclusions were confined to cytoplasmatic areas of adipohemocytes whereas other ones were relatively free of organelles. Due to their scarcity within the hemocyte population, oenocytes were not easy to spot under TEM, although cells displaying a characteristic uniformly dense cytoplasm and a paucity of organelles, compatible with their description were observed (Figure 5F). Oenocytes presented either centrally or eccentrically located nuclei with several patches of heterochromatin.

### 3.3. Fluorescence Microscopy

**Figure 6:**
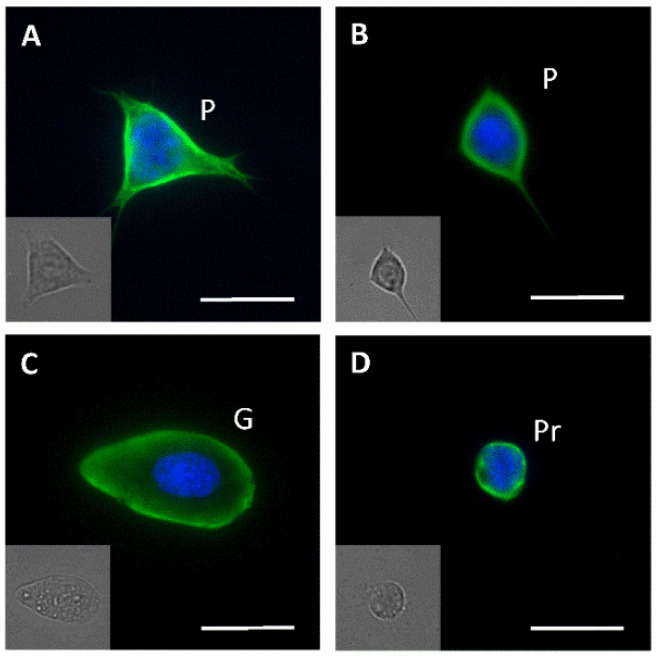
(**A**–**D**) Immunofluorescence microscopy of fifth instar nymphs hemocytes of *Dipetalogaster maxima* employing an anti-tubulin primary antibody. The insets show the corresponding differential interference contrast (DIC) images. The nuclei were stained with DAPI. P, plasmatocyte; G, granulocyte; Pr, prohemocyte. Bars: 10 μm.

Immunofluorescence using an anti-tubulin antibody applied to monolayers of hemocytes did not allow, by itself, to unequivocally identify all cell types present in the hemolymph. Nevertheless, together with the corresponding differential interference contrast (DIC) images, it was useful to detect cells compatible with plasmatocytes, granulocytes and prohemocytes (Figure 6). The putative plasmatocytes presented several pseudopodia and a flattened surface (Figure 6A,B), while the granulocytes displayed the typical rounded to oval shape and the presence of granules of a uniform size (Figure 6C). Putative prohemocytes presented their typical rounded shape and smaller diameter when compared to other cell types (Figure 6D).

### 3.4. Flow Cytometry Analysis

**Figure 7:**
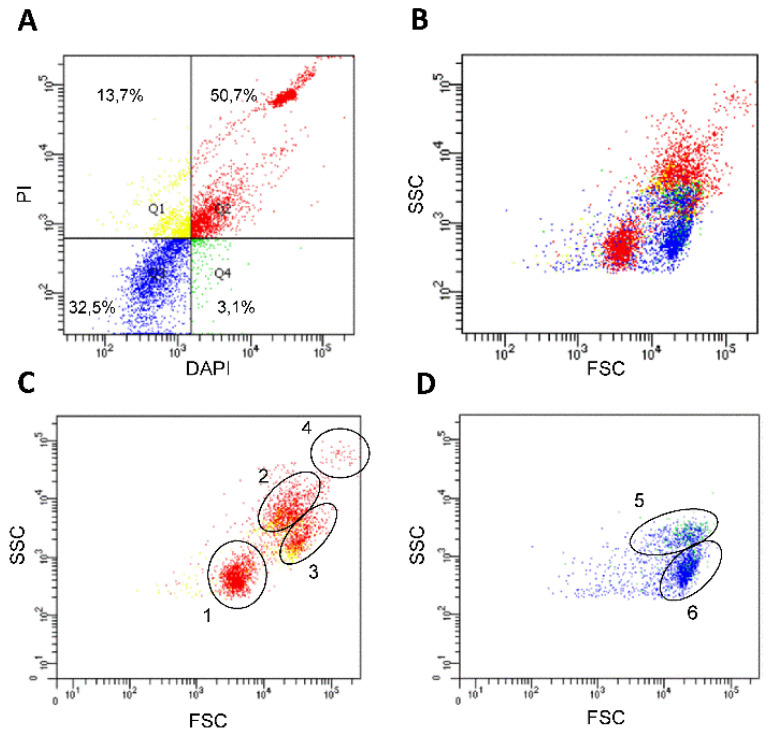
Hemocyte analysis by flow cytometry. Hemolymph samples were probed with PI and DAPI as described in Materials and Methods. (**A**) Dot-plot showing four groups as determined in comparison with controls employing only PI, DAPI or no fluorophore. (**B**) FSC vs SSC plot of the four groups displaying information of size and complexity, respectively. (**C**,**D**) are FSC vs SSC plots of Q1–Q2 and Q3–Q4 of (**A**) in matching colors. Numbers 1 to 6 are cell populations that can be grouped by size and complexity. The experiment was conducted by quintuplicated (n = 5) and each analyzed sample corresponded to the hemolymph collected from one insect. The panel shows the data of a representative experiment.

As a first attempt to employ a non-microscopy-based approach to differentiate hemolymph cell types, we used flow cytometry and the fluorescent nuclear markers PI and DAPI, which allowed to detect cells with different permeability (Figure 7). In combination with non-stained controls, the approach enabled us to differentiate four cell groups (Figure 7A, quadrants Q1–Q4). The larger one (50.7%), shown in the upper right quadrant (Q2), indicated the cells strongly stained with both markers. The percentages corresponding to the four cell groups were maintained among different biological replicates with a variation smaller than 15% (data not shown). As shown in Figure 7B–D, the cell populations were further characterized by size (FSC) and complexity (SSC), evidencing an important heterogeneity in the two larger groups displayed (Figure 7A, Q2 and Q3). In addition, based on cell size and complexity up to six cell populations could be grouped (Figure 7C,D).

## 4. Discussion

In all the insect species studied so far and particularly in triatomines, classification of hemocytes is a difficult issue because the main criterion currently employed is their morphology; however, it has to be considered that cell shapes might change according to the species in study, the developmental stage of the insect and the physiological status [3,4,35,36]. Moreover, the morphology of the hemocytes might be affected by the methodology employed in its study [20,23], and that is why sample collection and further processing and observation are crucial steps to be standardized. Previous works have reported four to seven types of hemocytes in triatomines, thus reflecting the difficulty of classification based solely on morphological characters. In *R. prolixus*, Wigglesworth initially described four different types and, using terminology taken from vertebrate hematology, they were named proleukocytes, oenocytoids, amoebocytes and lipocytes [24,26]. Later on, plasmatocytes and adipocytes were added to that classification [27,28]. In 1965, Jones [23] reclassified *R. prolixus*’ hemocytes also into six types named prohemocytes, plasmatocytes, granular hemocytes, oenocytoids, adipohemocytes and granulocytophagous cells. However, in 1970, Lai-Fook [37] identified only four hemocyte types in the same species. In the triatomine *Panstrongylus megistus*, Barracco et al. [21] also reported six hemocyte types. The first attempt to provide a way for comparison across species was carried out by Azambuja et al. [20], using six triatomine species, including *D. maxima*. These authors confirmed most of Jones’ classification [23] and recommended the use of sterile glassware to prevent cell lysis and deformation [20]. 

**Figure 8:**
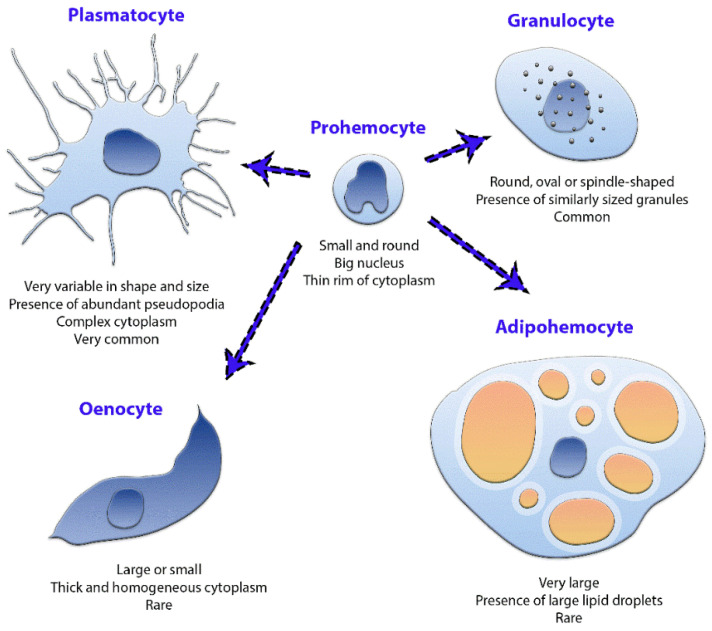
Schematic representation of the main features of each hemocyte type found in the hemolymph of fifth instar nymphs of *Dipetalogaster maxima*. The dashed arrows refer to the possibility that all cell types arise from the prohemocytes.

The classic works previously reported in triatomines agreed in that phase contrast of recently collected, unfixed and unstained cells was the best approach to identify the cell types present in the hemolymph of these species [20,21,23]. In samples of *D. maxima*’s fifth instar nymphs we were able to discriminate five cells types: the more abundant plasmatocytes and granulocytes as well as the less abundant prohemocytes, adipohemocytes and oenocytes (Figure 8); mainly in agreement with Azambuja et al. [20]. However, in our experimental conditions we did not observe cystocytes, which, according to Azambuja et al. [20], are very fragile cells similar to plasmatocytes and granulocytes but with larger granules. It is possible that these unstable cells were lost during pre-analytical procedures. In fact, in all preparations some degree of granulation and presence of debris was spotted. Nevertheless, and as suggested by the authors [20], cystocytes could be a subtype of plasmatocyte, which in turn could explain the difficulty in classifying them as different entities. Furthermore, Azambuja et al. [20] identified cystocytes and spindle cells in *D. maxima* but did not find adipohemocytes. The same authors recognized that spindle cells could be in fact granulocytes, plasmatocytes and/or oenocytes. 

In our experience and according to the literature [20,23], the use of phenylthiourea to avoid melanization in combination with an anticoagulant solution as well as sterile conditions for sample collection successfully stabilize the cells for proper observation. However, the use or the lack of sterile coverslips did not noticeably change the outcome. Even in our standardized experimental conditions, some islets of coagulation were observed, mainly in fresh, unfixed cells. This is probably explained by the contact of hemolymph with air at the time of sample collection. It is worth mentioning that direct comparison of cell morphology between samples disposed in monolayers and suspended cells, such as those observed by TEM, may be inaccurate. In our experimental conditions, the hemocytes analyzed by phase contrast microscopy and immunofluorescence assays were under similar technical conditions, allowing the cells enough time during the observation to decant, attach to the substrate, become flattened and project pseudopodia. Despite all these caveats, some cell types were distinguishable in all settings, being prohemocytes a clear example of this. These cells were small and round with a high ratio of nucleus/cytoplasm. Barraco and Loch [22] in *P. megistus*, observed intermediate forms between prohemocytes and plasmatocytes, supporting the hypothesis that prohemocytes act as stem cells and can originate the rest cell types. On the other hand, plasmatocytes spread relatively rapidly and showed numerous vacuoles and vesicles, RER and Golgi cisternae, which suggest a cell active in synthetic processes. They were the most numerous type in islets of aggregation, showing a wide variability in both shape and size. Their morphological variability could be related to the existence of cell subtypes and this highlights the need for specific markers. According to the literature, plasmatocytes are highly phagocytic and present numerous lysosomes, being the primary line of defense against microbial infection [38,39]. It is possible that giant cells are, in fact, plasmatocytes that have engulfed other cell types [1]. Granulocytes were very stable and small pseudopodia were present in some of them. It was proposed that granulocytes empty their content upon a foreign agent to release cytokines which in turn will attract plasmatocytes [38]. Some authors considered that cystocytes, also called coagulocytes, are a small form of granulocyte that participates in coagulation reactions [1]. Oenocytes presented a thick, homogeneous cytoplasm with few organelles and were also found in islets of aggregation. There are some reports proposing that oenocytes contain endogenous phenoloxidase activity and, therefore, participate in melanization, wound healing and encapsulation [20,38]. Adipohemocytes, with characteristic large lipid droplets displayed cytoplasmatic granules, which in association with RER and Golgi apparatus cisternae are indicative of synthetic and secretory cellular functions [1]. According to some authors, adipohemocytes might be fat body cells released to the circulation at the moment of hemolymph collection [20,23]. 

Few works have performed the ultrastructural characterization of triatomine hemocytes, including Lai-Fook [37] in adults of *R. prolixus* and Barracco et al. [22] in nymphs of *P. megistus*. Lai-Fook [37] analyzed the morphology and fine structure in an attempt to correlate these characteristics with function and identified four ultrastructural types of hemocytes; our findings in a general view, match the previous description of putative prohemocytes, plasmatocytes, oenocytes and granulocytes. In *P. megistus*, Barracco et al. [22] identified six hemocyte types, a fact which is also in agreement with most of our findings in terms of general morphology. Nevertheless, two main differences were found when comparing the hemocytes of *P. megistus* with those of *D. maxima*. Thus, in the latter species, coagulocytes were not found and the prohemocytes were relatively richer in organelles. 

Flow cytometry was employed in bees in an attempt to refine the classification of hemocytes and to better understand the functions of different subtypes [40]. However, in triatomines and other insect species, flow cytometry was used mainly to assess DNA content [41,42]. In *D. maxima*, the results obtained by flow cytometry employing fluorescent nuclear markers, combined with cell size and complexity, led us to recognize hemocyte populations, supporting a proof-of-concept that such a technique can be applied to objectively separate the hemocyte populations in triatomines. Based on size and the morphological features described above, we expected that prohemocytes and the smaller plasmatocytes were found in the flow cytometry population 1, granulocytes and larger plasmatocytes in populations 2, 3 and 5 and oenocytes in population 6, whereas adipohemocytes and giant cells could be part of cells located in population 4 (Figure 7C,D). Nevertheless, further experimental evidence will be necessary to validate this hypothesis. The use of other markers such as labeled wheat germ agglutinin (WGA), which is known to interact with the cell surface of triatomine hemocytes [30], as well as cell sorting in combination with light and electron microscopy would add even more discriminative power.

## 5. Conclusions

In the present work, we used phase contrast microscopy in unfixed cells to classify the hemocytes of fifth instar nymphs of the triatomine *D. maxima*. Thus, plasmatocytes, granulocytes, prohemocytes, adipohemocytes and oenocytes were identified and further characterized by immunofluorescence, flow cytometry and transmission electron microscopy. The main features of each cell type are depicted in Figure 8. Our findings shed light upon hemocyte classification in triatomines, a subject that has been a matter of controversy since pioneering studies [23,24,25,26,27,28]. This work paves the way for future investigations which hopefully might contribute to hemocyte classification employing other complementary approaches such as flow cytometry and cell sorting. This knowledge can be applied to unravel the hemocyte physiological functions in insect immune responses, coagulation cascades and endocrine processes, among the different events which involve these multifunctional and fascinating cells.

## Data Availability

Not applicable.
